# Supplementary material for: Causal interplay between lactose intolerance and gut microbiota: a combined bidirectional Mendelian randomization and in vivo validation study
Source: Front Nutr. 2026 Jun 1;13:1803337. doi: 10.3389/fnut.2026.1803337 (PMC13265576; doi:10.3389/fnut.2026.1803337)
Supplement: Supplementary file 1 [file Data_Sheet_1.zip › supplementary materials/Forward/leave one out cross validation/ebi-a-GCST90027453.finngen_R12_E4_LACTONAS.pdf]

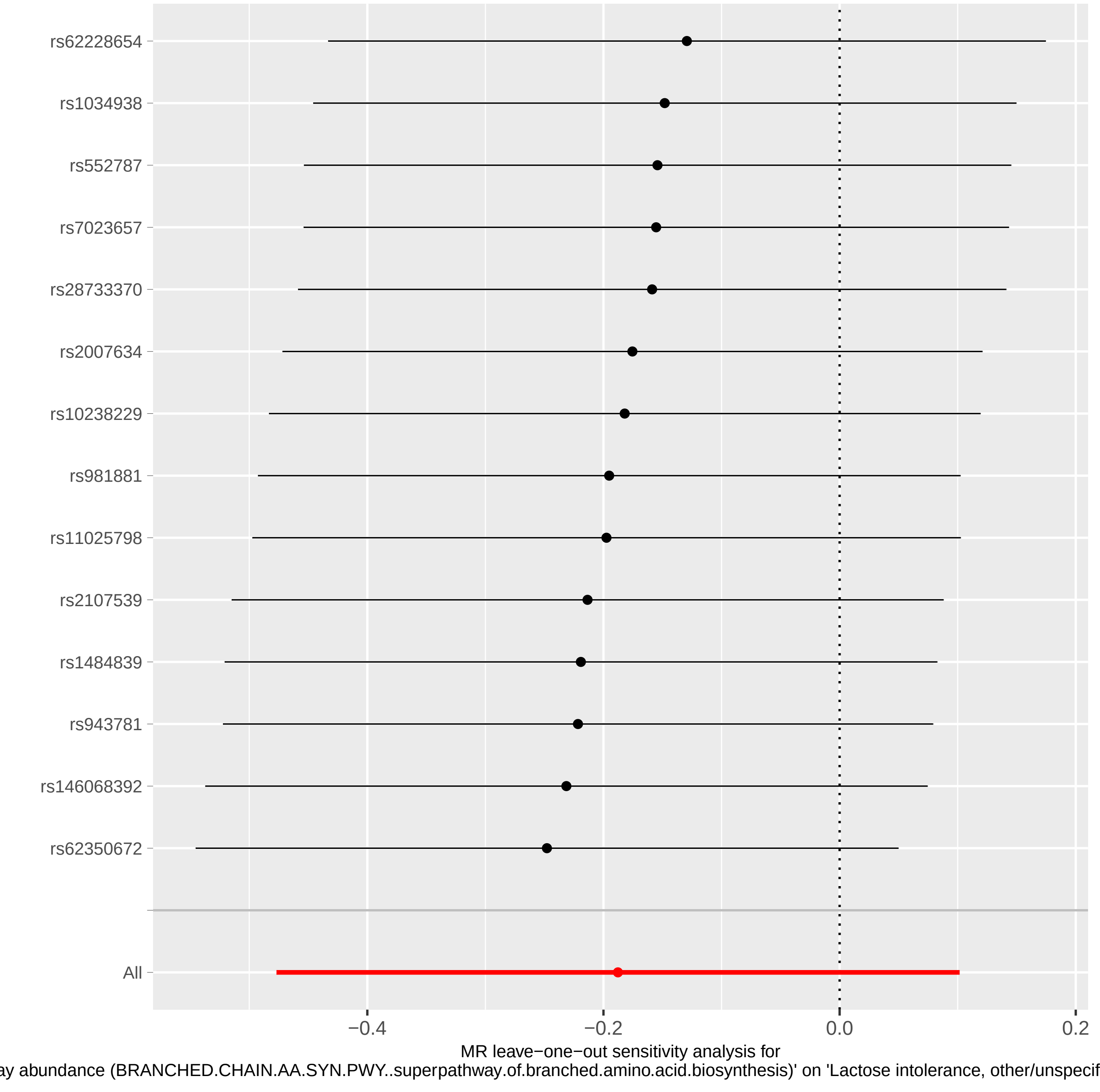
rs62228654
rs1034938
rs552787
rs7023657
rs28733370
rs2007634
rs10238229
rs981881
rs11025798
rs2107539
rs1484839
rs943781
rs146068392
rs62350672
All
−0.4
−0.2
0.0
0.2
MR leave−one−out sensitivity analysis for
ay abundance (BRANCHED.CHAIN.AA.SYN.PWY..superpathway.of.branched.amino.acid.biosynthesis)' on 'Lactose intolerance, other/unspecif
